# Intensive versus standard blood pressure control and overall brain small vessel disease burden: a post-hoc analysis of the SPRINT randomized clinical trial

**DOI:** 10.1016/j.eclinm.2026.104143

**Published:** 2026-08-11

**Authors:** Sokratis Charisis, Nicholas M. Pajewski, Larry R. Price, Ngoc Huynh Ho, Tanweer Rashid, David Wang, Yuheng Zeng, Niyas Shamsudeen Kutty, R. Nick Bryan, Kyle C. Kern, Bradford C. Dickerson, Sudha Seshadri, Ilya Nasrallah, Lenore Launer, Christos Davatzikos, Jeff D. Williamson, Mohamad Habes

**Affiliations:** a Neuroimage Analytics Laboratory (NAL) and the Biggs Institute Neuroimaging Core (BINC), Glenn Biggs Institute for Alzheimer's and Neurodegenerative Diseases, University of Texas Health Science Center at San Antonio, San Antonio, TX, USA; b Department of Neurology, Massachusetts General Hospital, Boston, MA, USA; c Harvard Medical School, Boston, MA, USA; d Department of Biostatistics and Data Science, Wake Forest University School of Medicine, Winston-Salem, NC, USA; e Department of Psychiatry and Behavioral Sciences, University of Texas Health Science Center at San Antonio, San Antonio, TX, USA; f Department of Radiology, Perelman School of Medicine, University of Pennsylvania, Philadelphia, PA, USA; g Department of Neurology, UCLA David Geffen School of Medicine, Los Angeles, CA, USA; h Neuroepidemiology Section, Intramural Research Program, National Institute on Aging, Bethesda, MD, USA; i Section of Gerontology and Geriatric Medicine, Department of Internal Medicine, Wake Forest University School of Medicine, Winston-Salem, NC, USA

**Keywords:** Cerebral small vessel disease, Intensive blood pressure treatment, Dose–response relationship, Randomized clinical trial, Brain MRI, Neuroimaging biomarkers, SPRINT

## Abstract

**Background:**

The benefits of improved systolic blood pressure (SBP) control on stroke, coronary heart disease, and heart failure are well-established, yet its effect on overall cerebral small vessel disease (SVD) burden remains uncharacterized. We examined the association between intensive SBP control and change in SVD burden.

**Methods:**

We conducted a post-hoc analysis of the Systolic Blood Pressure Intervention Trial (SPRINT), a multicenter randomized clinical trial. Of 1267 hypertensive individuals aged ≥50 years without diabetes or prior stroke screened for the brain MRI substudy, 663 and 442 participants completed brain MRI that met quality control criteria and had complete data on SVD indicators at baseline and at a median of 3.9 (interquartile range, 3.6–4.1) years after randomization, respectively. From November 2010 to March 2013, participants were randomly assigned to an intensive SBP target of <120 mmHg (n = 348) or a standard target of <140 mmHg (n = 315). Post-hoc outcome was change in a global SVD factor, longitudinally validated using confirmatory factor analysis and designed to capture overall SVD-related vascular brain injury by integrating three complementary imaging endophenotypes: periventricular white matter hyperintensities, white matter free water, and basal ganglia perivascular spaces. This trial is registered with ClinicalTrials.gov (NCT01206062).

**Findings:**

Mean [SD] baseline age was 68.1 (8.6) years; 263 [40%] participants were women. Compared with standard SBP treatment, intensive treatment was associated with significantly less SVD progression (standardized mean difference [Cohen's d] = −0.40 [95% CI, −0.62 to −0.17]). We also observed gradually more favorable SVD burden changes with greater attained SBP reductions, demonstrating a clear dose–response relationship: 21.2% (95% CI, 7.4%–35%), 26.3% (13.1%–39.5%), and 39.4% (24.2%–54.5%) less progression relative to baseline SVD burden for SBP reductions of 0–10 mmHg, 10–20 mmHg, and ≥20 mmHg, respectively.

**Interpretation:**

Among hypertensive adults, targeting an SBP of <120 mmHg, compared with <140 mmHg, was associated with less progression of SVD burden. Even modest SBP reductions of ≤10 mmHg conferred measurable brain benefits, with larger reductions providing incrementally greater protection against SVD progression.

**Funding:**

National Institutes of Health, National Heart, Lung, and Blood Institute, National Institute of Diabetes and Digestive and Kidney Diseases, National Institute on Aging, and National Institute of Neurological Disorders and Stroke.

**Research in context**

**Evidence before this study**

We searched PubMed from inception to January 30, 2026, for studies examining the effects of blood pressure lowering on cerebral small vessel disease (SVD), using combinations of the terms “cerebral small vessel disease”, “white matter hyperintensities”, “perivascular spaces”, “diffusion MRI”, and “blood pressure”. We focused on randomized clinical trials and major observational or meta-analytic studies evaluating associations of blood pressure lowering with cardiovascular outcomes, mortality, or MRI markers of SVD. Although prior evidence strongly supports the benefits of improved systolic blood pressure (SBP) control for all-cause mortality and cardiovascular outcomes, including stroke, major cardiovascular events, coronary heart disease, and heart failure, its effect on overall brain SVD burden has not been fully characterized. In addition, while prior interventional and observational studies have demonstrated that the benefits of SBP lowering on major cardiovascular outcomes, vascular mortality, and all-cause mortality are proportional to the magnitude of the achieved SBP reduction, it is unknown whether a similar dose–response relationship exists for SVD burden. Establishing such a relationship could provide crucial biological and clinical insights and inform optimal SBP targets for preserving brain health.

**Added value of this study**

Using longitudinal MRI data from the Systolic Blood Pressure Intervention Trial (SPRINT), a multicenter randomized clinical trial, this post-hoc analysis applied a biology-informed biomarker framework designed to capture overall SVD-related vascular brain injury by integrating multiple complementary MRI endophenotypes: periventricular white matter hyperintensities, basal ganglia perivascular spaces, and white matter free water. Among hypertensive adults aged 50 years or older without diabetes or prior stroke, targeting an SBP of <120 mmHg, compared with <140 mmHg, was associated with significantly less progression of overall SVD burden. Notably, we also observed a clear dose–response relationship between attained SBP reduction and longitudinal change in SVD burden, supporting a biologically coherent link between hemodynamic exposure and microvascular brain injury. Even modest SBP reductions of ≤10 mmHg were associated with measurable brain benefits, while larger reductions were associated with progressively less SVD progression.

**Implications of all the available evidence**

Clinical manifestations of SVD are broad and include stroke, cognitive impairment, gait and balance problems, mood disorders, and extrapyramidal symptoms, contributing substantially to disability, reduced quality of life, and mortality. Existing clinical trial evidence supports an SBP goal of <130 mmHg and, when feasible, <120 mmHg for cardiovascular disease risk reduction. This study provides evidence that the benefits of lower SBP goals may extend to SVD burden and that the magnitude of attained SBP reduction may be relevant. Incremental decreases in SBP were associated with gradually more favorable changes in SVD burden, while even modest SBP reductions of ≤10 mmHg might confer some benefit in slowing SVD progression for individuals unable to tolerate more intensive BP lowering. Future studies should examine the generalizability of these findings to hypertensive populations not represented in SPRINT, including individuals with diabetes, prior stroke, dementia, advanced kidney disease, or nursing home residence.

## Introduction

Cerebral small vessel disease (SVD) accounts for up to 25% of ischemic strokes and is the leading vascular contributor to cognitive decline and dementia.^1^ Although the benefits of blood pressure (BP) lowering on all-cause mortality and cardiovascular outcomes such as stroke, major cardiovascular events, coronary heart disease, and heart failure are well-established,^2^^,^^3^ its effect on overall SVD burden has not been fully characterized. In addition, while prior interventional and observational studies have shown that the benefits of BP lowering on major cardiovascular outcomes, vascular mortality, and all-cause mortality are proportional to the magnitude of the achieved BP reduction,^2^^,^^4^ it is unknown whether a similar dose–response relationship exists for SVD burden. Establishing whether such a relationship exists could provide crucial biological and clinical insights, as dose–response effects are a hallmark of causal vascular mechanisms and may inform optimal BP targets for preserving brain health.

Previous clinical trials have indicated less progression of white matter hyperintensity (WMH) volume, an imaging endophenotype of SVD, with more intensive BP control.^5^, ^6^, ^7^, ^8^, ^9^ However, MRI markers traditionally considered to represent SVD—including WMH, perivascular spaces (PVS),^10^ and measures of white matter microstructural damage^11^—may also reflect other underlying processes, such as brain aging or neurodegeneration,^12^, ^13^, ^14^, ^15^, ^16^ making it difficult to distinguish the extent to which observed associations can be attributed to SVD versus the effects of BP on other biological processes. Moreover, these individual markers may each capture only certain aspects of SVD rather than its global burden.^1^^,^^17^^,^^18^ To address these challenges, we previously introduced a latent variable analytical framework that integrates multiple complementary MRI markers in strategic brain regions to more specifically represent the SVD dimension and more accurately capture the total SVD burden.^18^

We leveraged this analytical framework to examine the effect of intensive versus standard BP control on SVD burden in the Systolic Blood Pressure Intervention Trial (SPRINT), a multicenter randomized clinical trial of hypertensive individuals aged ≥50 years without a history of diabetes or stroke.^19^ We studied the effect of treatment assignment on SVD burden, but also examined the association between incremental attained reductions in systolic BP (SBP) and SVD burden change.

## Methods

### Trial design and participants

We conducted a post-hoc analysis of the SPRINT trial. The trial design and primary endpoint have been previously described,^19^^,^^20^ and the trial protocol is provided in Supplement 1. Eligibility criteria included age ≥50 years, SBP 130–180 mmHg at the screening visit, and increased cardiovascular risk—defined as prevalent clinical or subclinical cardiovascular disease, chronic kidney disease (estimated glomerular filtration rate of <60 mL/min/1.73 m^2^), a 10-year Framingham cardiovascular disease risk score of ≥15%, or age ≥75 years. Exclusion criteria included nursing home residency, dementia diagnosis (based on medical record review) or treatment with medications primarily used for dementia, prevalent diabetes, or prior stroke. From November 2010 to March 2013, 9361 participants were randomized in a 1:1 allocation to an intensive treatment strategy with SBP goal of <120 mmHg or a standard treatment strategy with SBP goal of <140 mmHg.^20^ At randomization, a subset of participants (n = 1267) were invited to undergo brain MRI at baseline and 48-month follow-up, at one of seven designated MRI sites (across 27 clinical sites). Enrollment in the MRI substudy required accessibility to a designated MRI site. Exclusion criteria included presence of pacemaker, defibrillator, neurostimulator or other implanted electrical device; ferromagnetic or unknown cerebral aneurysm clip; cochlear or other otologic implant; unknown metallic foreign bodies or a history of metal fragment exposure in or around the eyes; and severe claustrophobia.

Participants who completed both baseline and follow-up MRI scans, met MRI quality control criteria, and had complete data on SVD indicators were included in this analysis.

### Ethics statement

The study was approved by the institutional review board at each participating site and adhered to the Declaration of Helsinki. All participants provided written informed consent. This trial is registered with ClinicalTrials.gov (NCT01206062).

### Measurements

Baseline age and sex were collected via self-reporting. Information on race and ethnicity was collected using fixed categories from the Policy and Guidelines on The Inclusion of Women and Minorities as Subjects in Clinical Research issued by the National Institutes of Health.

BP measurements were obtained with the Professional Digital Blood Pressure Monitor (Omron Healthcare; model 907XL) using standardized techniques.^21^ A total of three seated automated BP measurements were obtained and their mean was documented as visit BP. BP measurement training emphasized proper positioning of participants, measurement of arm circumference and use of proper cuff size, and a 5-min rest period before obtaining each of the three measurements. During the rest and BP measurement periods, the participant abstained from questionnaire completion, talking, or texting.

### Procedures

The SPRINT trial procedures are fully described in the study protocol (Supplement 1). All major classes of antihypertensive agents were included in the trial formulary and provided to participants at no cost. After randomization, the baseline antihypertensive regimens of study participants were adjusted according to the treatment assignment. Dose adjustments were based on visit BP.

### Brain MRI acquisition, scanning protocol, and quality control

Brain MRI scans were acquired at seven designated MRI sites with different scanner models (Supplemental Table S1). Scanner performance was monitored with quarterly quality checks using the Alzheimer's Disease Neuroimaging Initiative and Function Biomedical Informatics Research Network phantom scans, with all scanners showing stability of phantom measurements throughout the study period.

Scanning sequences included T1-weighted, T2-weighted, fluid-attenuated inversion recovery (FLAIR), pseudo-continuous arterial spin labeling (PCASL), and diffusion tensor imaging (DTI). Acquisition parameters are presented in Supplemental Table S2.

**Fig. 1:**
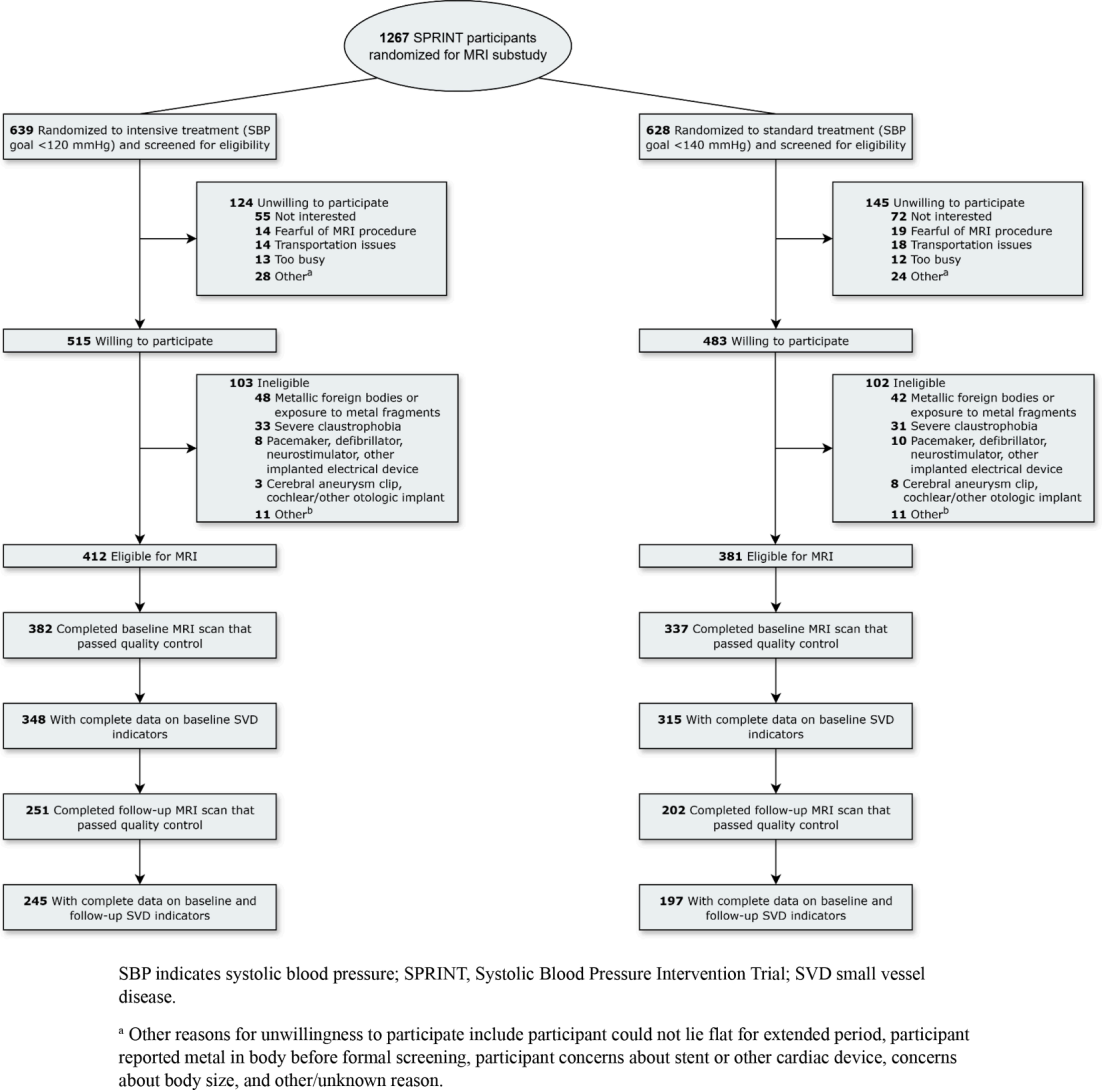
Flowchart of randomization, eligibility, and follow-up of participants in the Systolic Blood Pressure Intervention Trial (SPRINT) MRI substudy.

Images were visually inspected for distortions and motion artifacts; poor quality scans were excluded from the final analytic sample (Fig. 1).

### Brain MRI processing and SVD indicator measurements

Image analysts were blinded to the randomization assignment. An automated preprocessing pipeline including inhomogeneity correction^22^ and extraction of the intracranial tissues and cerebrospinal fluid using multi-atlas skull stripping^23^ was applied. Anatomical regions of interest (ROIs) were identified using a multi-atlas label fusion method to segment gray matter (GM) and white matter (WM) volumes (in mm^3^).^24^ The total intracranial volume (ICV) was calculated as the sum of GM, WM, and cerebrospinal fluid volumes.

#### WMH measurements

The volume of WMH was measured from inhomogeneity-corrected and co-registered FLAIR and T1-weighted images using a deep learning-based method^5^^,^^8^^,^^25^; WMH were grouped into periventricular and subcortical, as described elsewhere.^26^^,^^27^

#### PVS measurements

Quantification of PVS counts was performed from T2-weighted, T1-weighted, and FLAIR images using a deep learning-based method^18^^,^^28^^,^^29^; PVS were grouped into six distinct anatomic regions including basal ganglia, thalamus, insular, brainstem, frontoparietal, and temporal, as previously described.^18^^,^^29^

#### WM integrity measurements

Although we previously relied on conventional DTI indices such as mean WM fractional anisotropy and trace to capture the WM microstructural damage component of SVD,^1^^,^^10^^,^^11^ these measures are likely to be contaminated by extracellular water.^30^ To address this limitation and increase the robustness of the SVD indicators, we calculated mean WM free water (expressing the fraction of the diffusion signal explained by isotropically unrestricted water) using a validated imaging biomarker toolkit.^31^ WM free water is a sensitive biomarker of subtle brain injury related to vascular risk factors, and has demonstrated excellent instrumental properties based on a multi-site validation study assessing its inter-rater reliability, test-retest repeatability, and inter-scanner reproducibility.^31^

### Duration of follow-up

On August 20, 2015, the director of the National Heart, Lung, and Blood Institute accepted the SPRINT data and safety monitoring board's recommendation to inform investigators and participants of the cardiovascular outcome results and initiated the process to end the BP intervention. Consequently, most follow-up MRI scans included in this analysis (n = 424; 95.9%) were completed during the closeout period (August 20, 2015–July 27, 2016). During this period, participants were either receiving BP management by their primary care providers or were in the process of transitioning to this care, although antihypertensive medications were still being provided by the study.

### Outcome

The post-hoc outcome was change in a global SVD factor designed to capture overall SVD-related vascular brain injury by integrating three complementary MRI indicators: periventricular WMH, WM free water, and basal ganglia PVS.

### Statistical analysis

Differences between baseline and follow-up clinical and MRI measures were assessed using paired t-tests or Wilcoxon signed-rank tests for normally and non-normally distributed data, respectively.

To facilitate numerical stability and convergence of the structural equation modeling (SEM) algorithms, observed variables underwent appropriate rescaling such that their variances differed by no more than one order of magnitude. For variables measured at both baseline and follow-up, the same rescaling factor was applied.

#### SVD measurement model and invariance testing strategy

We used longitudinal confirmatory factor analysis (LCFA) models to test our previously developed measurement framework for SVD in SPRINT.^18^ In our original approach, the SVD measurement model included PVS in the basal ganglia and thalamus, WMH in periventricular and subcortical regions, and DTI indices of WM fractional anisotropy and trace as indicators. We sought to further optimize the measurement model by replacing traditional DTI measures with the more robust WM free water and using only the most reliable indicator (i.e., with the highest loading) from each pair of WMH and PVS markers to increase model parsimony. Therefore, SVD was modeled as a latent construct measured by periventricular WMH, basal ganglia PVS, and WM free water at two timepoints (baseline and follow-up). Measurement residuals for indicators expressing the same MRI marker across time were allowed to covary to account for shared indicator-specific variance.^32^

To ensure the SVD latent construct means could be meaningfully compared across the two timepoints, we evaluated the measurement properties of the MRI indicators over time (Supplemental Methods 1). We used the commonly accepted criterion of comparative fit index change (ΔCFI) < −0.01 to determine whether the null hypothesis of invariance should be rejected.^33^ In cases where measurement invariance was not supported, we used Multiple Indicators Multiple Causes (MIMIC) models to examine conceptually plausible sources of differential item functioning (DIF) over time.

#### Effect of treatment assignment on SVD burden

We used multiple-indicator Latent Change Score (LCS) models to examine the effect of treatment assignment on SVD burden (Supplemental Methods 2). In cases of DIF, we modified the LCS model by including regressions of the MRI indicators on covariates accounting for DIF, conditioning the measurement model on plausible values of these covariates. The structural part of the model included a regression of the latent change score on treatment assignment to estimate the effect of the latter on SVD burden change. The standardized mean difference between the intensive and standard BP treatment groups (Cohen's d) was also computed within the LCS model by dividing the treatment effect estimate by the square root of the latent change score variance (i.e., model-implied pooled standard deviation).

#### Attained SBP reduction and SVD change

We examined the association between attained reduction in SBP and SVD burden change by regressing the latent change score on a set of dummy variables representing ordinal categories of SBP reduction (0–10 mmHg, 10–20 mmHg, and ≥20 mmHg) from baseline. Attained SBP reduction was calculated from the average SBP across the 6-, 9-, 12-, 15-, and 18-month follow-up visits, relative to baseline. These follow-up time points were selected because, at the group level, BP generally stabilized after the first 6 months of trial follow-up.

#### BP mediation of the treatment effect

To determine whether the observed treatment effect on SVD burden was driven by intervention-related reductions in SBP, or by other factors unrelated to SBP change, we examined the mediating effect of SBP change in the relationship between treatment assignment and SVD burden change.

#### Sensitivity analyses

To test the robustness and stability of our findings, we conducted the following set of sensitivity analyses: (i) we adjusted the LCS models for baseline age, sex, race, and ICV; (ii) we repeated the analyses using ROI volume-normalized periventricular WMH and basal ganglia PVS measures to account for potential volumetric differences between baseline and follow-up scans that could have influenced our results; (iii) we included all participants with available baseline MRI indicator data using full information maximum likelihood with auxiliary variables for handling missing follow-up MRI indicator data (Supplemental Methods 3) to examine whether differential losses to follow-up—given the lower than expected MRI completion rate in the SPRINT MRI substudy^5^^,^^8^^,^^34^—may have affected our findings.

All models were computed using the lavaan package.^35^ Parameter estimates were computed using the maximum likelihood estimator. Robust (Huber-White) standard errors and a scaled (Yuan-Bentler) test statistic were computed to account for potential violations of normality. For mediation models, bias-corrected 95% CIs for indirect and direct effects were calculated using 5000 bootstrap samples.

Analyses and graphics were performed using R, version 4.4.3 (R Foundation for Statistical Computing, 2025). A two-sided P value of <0.05 was considered statistically significant.

### Role of the funding source

The funder of the study had no role in study design, data collection, data analysis, data interpretation, or writing of the report.

## Results

### Participant characteristics

Baseline participant characteristics by MRI substudy enrollment are presented in Supplemental Table S3. Of the 1267 SPRINT participants screened for the MRI substudy, 998 were willing to participate, 793 were eligible, 719 completed a baseline MRI scan that passed quality control, and 663 had complete data on baseline SVD indicators (Fig. 1). These 663 participants had a mean (SD) age of 68.1 (8.6) years, and 263 (40%) were women. Among them, 315 (48%) were randomized to standard BP treatment and 348 (52%) to intensive BP treatment (Table 1).

**Table 1:** Participant baseline characteristics by treatment assignment.

	Completed baseline MRI scan (N = 663)		Completed baseline and follow-up MRI scans (N = 442)	
	Standard treatment (n = 315)	Intensive treatment (n = 348)	Standard treatment (n = 197)	Intensive treatment (n = 245)
Demographic and vascular characteristics				
Age, mean (SD), years	67.6 (8.7)	68.5 (8.6)	67.4 (8.3)	68.8 (8.5)
Sex, n (%)				
Men	202 (64%)	198 (57%)	132 (67%)	148 (60%)
Women	113 (36%)	150 (43%)	65 (33%)	97 (40%)
Race/ethnicity, n (%)				
Black	99 (31%)	112 (32%)	57 (29%)	66 (27%)
Hispanic	21 (6.7%)	13 (3.7%)	13 (6.6%)	8 (3.3%)
White	190 (60%)	218 (63%)	123 (62%)	168 (69%)
Other	5 (1.6%)	5 (1.4%)	4 (2.0%)	3 (1.2%)
Fasting HDL cholesterol, mean (SD), mg/dL	53.1 (14.6)	53.6 (14.7)	53.4 (16.2)	53.3 (15.1)
Body mass index, mean (SD), kg/m^2^	29.8 (5.3)	29.6 (5.3)	29.3 (4.8)	29.4 (5.1)
Systolic blood pressure, mean (SD), mmHg	138.4 (15.2)	138.3 (17.5)	138.8 (15.5)	136.2 (16.8)
Diastolic blood pressure, mean (SD), mmHg	78.1 (12.1)	77.0 (11.2)	78.6 (12.3)	76.3 (10.8)
MRI-derived volumes				
Intracranial volume, mean (SD), mm^3^	1,408,110 (137,125)	1,390,369 (139,602)	1,419,003 (137,492)	1,400,771 (137,382)
Periventricular WM ROI, mean (SD), mm^3^	119,892.2 (21,203.0)	120,758.1 (21,783.7)	119,911.8 (21,302.0)	120,801.6 (21,809.6)
Basal ganglia ROI, mean (SD), mm^3^	26,990.5 (2829.8)	26,900.3 (2998.8)	27,152.9 (2894.6)	26,932.9 (3012.6)
SVD indicators				
Periventricular WMH, median (IQR), mm^3^	2413.0 (1097.8, 4508.1)	2267.7 (1030.4, 4915.6)	2437.0 (1186.0, 4514.0)	2183.9 (961.3, 4780.8)
Mean WM free water, mean (SD)	0.234 (0.040)	0.236 (0.041)	0.232 (0.038)	0.233 (0.040)
Basal ganglia PVS, mean (SD), count	94.9 (35.8)	99.6 (37.4)	94.7 (34.4)	98.6 (37.9)
ROI volume-normalized SVD indicators				
Periventricular WMH, median (IQR), mm^3^/mm^3^	0.021 (0.010, 0.036)	0.019 (0.010, 0.043)	0.021 (0.011, 0.036)	0.018 (0.009, 0.039)
Basal ganglia PVS, mean (SD), count/mm^3^	0.003 (0.001)	0.004 (0.001)	0.003 (0.001)	0.004 (0.001)

Abbreviations: HDL, high-density lipoprotein; WM, white matter; ROI, region of interest; SVD, small vessel disease; WMH, white matter hyperintensities; PVS, perivascular spaces.

Of the 663 participants with complete data on baseline SVD indicators, 453 completed a follow-up MRI scan that passed quality control, and 442 had complete data on both baseline and follow-up SVD indicators (Fig. 1). These 442 participants had a mean (SD) age of 68.2 (8.4) years, and 162 (37%) were women. Among them, 197 (45%) were assigned to standard BP treatment and 245 (55%) to intensive BP treatment (Table 1). The median time interval between baseline and follow-up MRI scans was 3.9 (interquartile range, 3.6–4.1) years. There was a significant reduction in SBP in both treatment groups (Table 2). Both treatment groups experienced an increase in periventricular WMH volume and mean WM free water. The intensive treatment group experienced a decrease in basal ganglia PVS count, whereas no significant change was observed in the standard treatment group.

**Table 2:** Baseline and follow-up study measures by treatment assignment.

	Standard treatment (n = 197)			Intensive treatment (n = 245)		
	Baseline	Follow-up	P value^a^	Baseline	Follow-up	P value^a^
Systolic blood pressure, mean (SD), mmHg	138.8 (15.5)	135.6 (7.6)	0.002	136.2 (16.8)	120.2 (8.6)	<0.001
MRI-derived volumes						
Intracranial volume, mean (SD), mm^3^	1,419,003 (137,492)	1,419,881 (140,083)	0.53	1,400,771 (137,382)	1,401,803 (137,278)	0.37
Periventricular WM ROI, mean (SD), mm^3^	119,911.8 (21,302.0)	124,072.2 (22,013.6)	<0.001	120,801.6 (21,809.6)	124,399.1 (22,439.4)	<0.001
Basal ganglia ROI, mean (SD), mm^3^	27,152.9 (2894.6)	26,754.2 (2919.0)	<0.001	26,932.9 (3012.6)	26,406.1 (2995.5)	<0.001
SVD indicators						
Periventricular WMH, median (IQR), mm^3^	2437.0 (1186.0, 4514.0)	3111.8 (1405.7, 6194.1)	<0.001	2183.9 (961.3, 4780.8)	2484.5 (1040.5, 5768.8)	<0.001
Mean WM free water, mean (SD)	0.232 (0.038)	0.246 (0.043)	<0.001	0.233 (0.040)	0.247 (0.044)	<0.001
Basal ganglia PVS, mean (SD), count	94.7 (34.4)	93.4 (35.9)	0.41	98.6 (37.9)	90.6 (36.0)	<0.001
ROI volume-normalized SVD indicators						
Periventricular WMH, median (IQR), mm^3^/mm^3^	0.021 (0.011, 0.036)	0.025 (0.013, 0.050)	<0.001	0.018 (0.009, 0.039)	0.020 (0.009, 0.044)	<0.001
Basal ganglia PVS, mean (SD), count/mm^3^	0.003 (0.001)	0.003 (0.001)	0.94	0.004 (0.001)	0.003 (0.001)	<0.001

Abbreviations: WM, white matter; ROI, region of interest; SVD, small vessel disease; WMH, white matter hyperintensities; PVS, perivascular spaces.

a Derived using paired t-tests or Wilcoxon signed-rank tests for normally and non-normally distributed data, respectively.

### SVD measurement model and longitudinal measurement invariance

For sequential measurement invariance testing and DIF investigation see Supplemental Results 1 and Supplemental Figure S1. After conditioning the LCFA models on mean baseline age (68.2 years), both metric and scalar invariance were supported, as indicated by ΔCFI ≥ −0.01 relative to the configural invariance model (Supplemental Table S4). Parameter estimates for the covariance and mean structures of the conditional LCFA model are presented in Supplemental Tables S5 and S6, respectively.

### Effect of treatment assignment on SVD burden

**Fig. 2:**
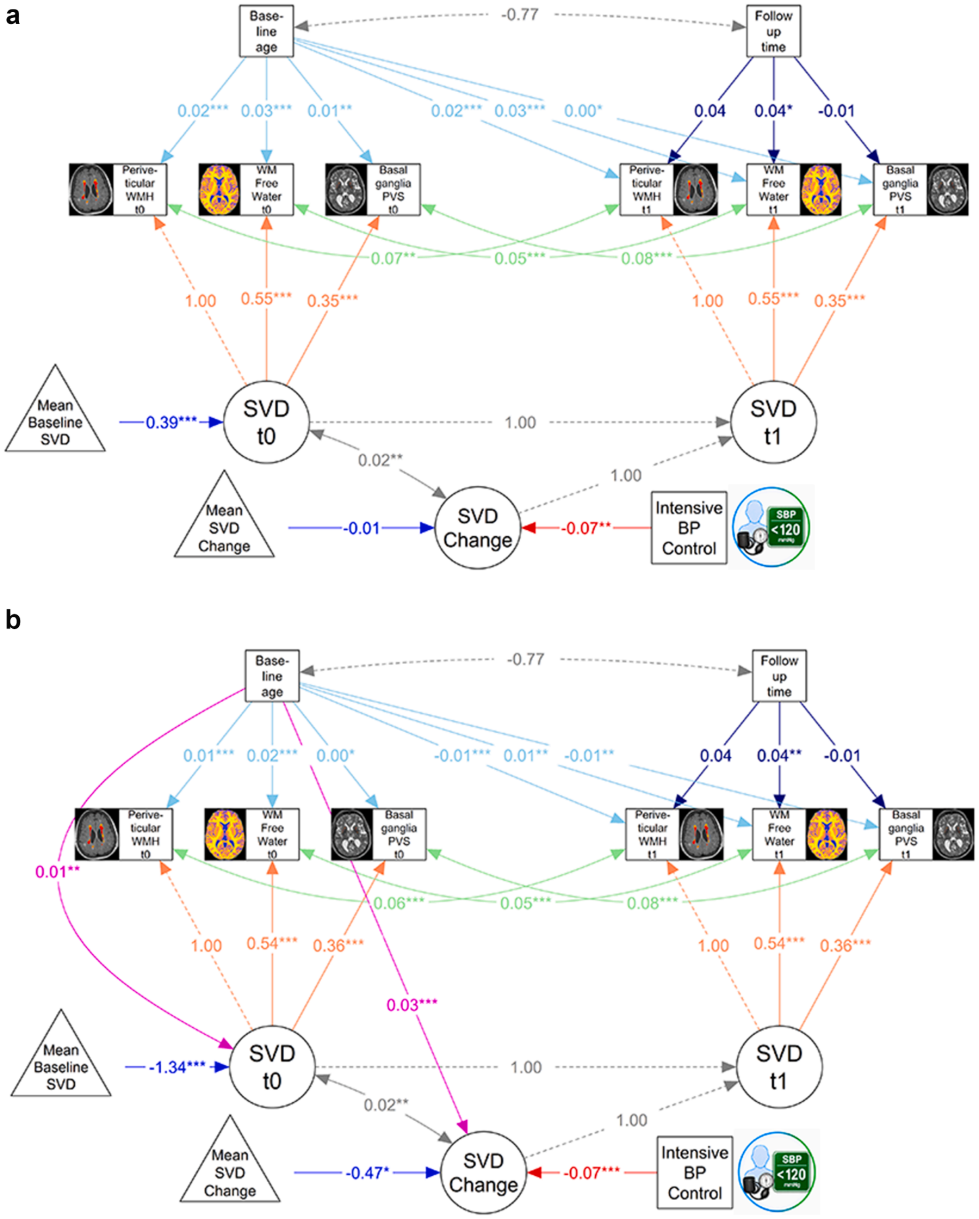
Multiple-indicator latent change score models examining the effect of treatment assignment on small vessel disease (SVD) burden. Panel a presents the unadjusted model, and panel b presents the model adjusted for baseline age, sex, race, and intracranial volume. SVD is modeled as a latent construct, measured by periventricular white matter hyperintensities (WMH), white matter (WM) free water, and basal ganglia perivascular spaces (PVS) at baseline (t0) and follow-up (t1). The model includes direct effects of baseline age and follow-up time on each MRI indicator to account for age-related differential item functioning. Latent SVD change is defined as the difference in SVD between t1 and t0. Intensive BP treatment group membership is modeled as a predictor of latent SVD change. Arrow values represent regression coefficients (or covariances for double-headed arrows). Asterisks indicate statistically significant parameter estimates at p < 0.05 (∗), p < 0.01 (∗∗), and p < 0.001 (∗∗∗).

The conditional (on age) LCS model revealed a significant association between intensive BP treatment assignment and latent SVD change, indicating that compared with standard BP treatment, intensive BP treatment was associated with a more favorable change in SVD burden (β = −0.066 [95% CI, −0.106 to −0.026], corresponding to 16.9% [6.9%–26.9%] less progression relative to mean baseline SVD burden, or a standardized mean difference [Cohen's d] of −0.395 [−0.616 to −0.174])—Fig. 2a. LCS models adjusted for age, sex, race, and ICV produced similar results (β = −0.07 [−0.11 to −0.03]; Cohen's d = −0.435 [−0.664 to −0.205])—Fig. 2b.

The LCS model with ROI volume-normalized periventricular WMH and basal ganglia PVS measures exhibited a similar association (β = −0.046 [−0.073 to −0.019]; Cohen's d = −0.405 [−0.629 to −0.182]), as did models including all individuals with available baseline MRI indicator data (N = 663) using full information maximum likelihood (β = −0.073 [−0.111 to −0.034]; Cohen's d = −0.42 [−0.63 to −0.21]). MRI examples illustrating SVD indicator progression in two representative participants—one from the standard BP treatment group with a high latent SVD change score, reflecting greater overall SVD burden progression, and one from the intensive BP treatment group with a low latent SVD change score, reflecting smaller overall SVD burden progression—are shown in Supplemental Figure S2.

### Attained SBP reduction and SVD change

**Fig. 3:**
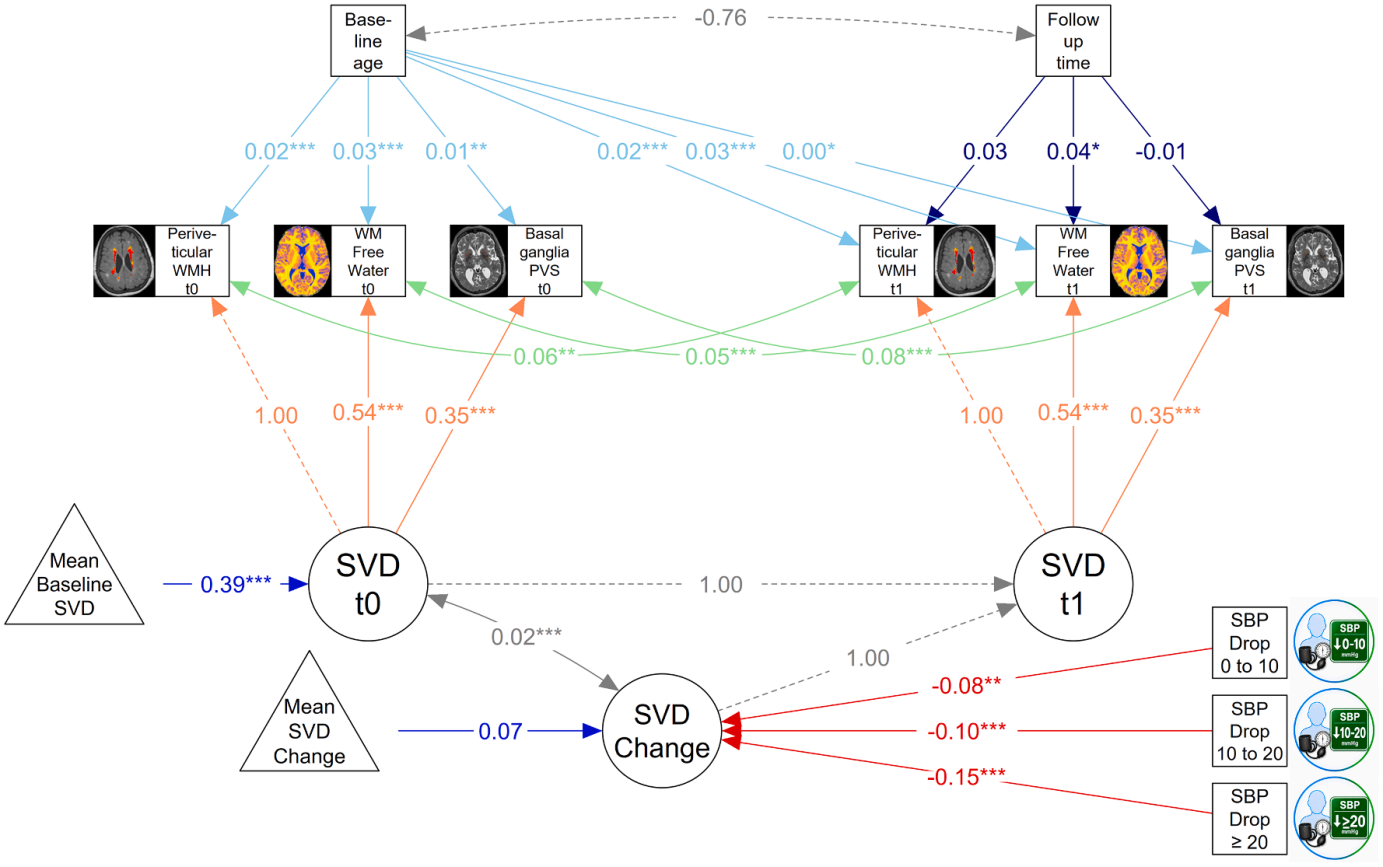
Multiple-indicator latent change score model examining the association between systolic blood pressure (SBP) reduction and change in small vessel disease (SVD) burden. SVD is modeled as a latent construct, measured by periventricular white matter hyperintensities (WMH), white matter (WM) free water, and basal ganglia perivascular spaces (PVS) at baseline (t0) and follow-up (t1). The model includes direct effects of baseline age and follow-up time on each MRI indicator to account for age-related differential item functioning. Latent SVD change is defined as the difference in SVD between t1 and t0. Dummy variables representing incremental SBP reductions from baseline are modeled as predictors of latent SVD change, with SBP increase as the reference group. Arrow values represent regression coefficients (or covariances for double-headed arrows). Asterisks indicate statistically significant parameter estimates at p < 0.05 (∗), p < 0.01 (∗∗), and p < 0.001 (∗∗∗).

Baseline participant characteristics across attained SBP groups are presented in Supplemental Table S7. In models examining the association between attained SBP reduction and SVD change, compared with participants who experienced an increase in SBP (n = 118), those with progressively greater attained SBP reductions exhibited gradually more favorable changes in SVD burden (β_0–10mmHg_ = −0.083 [−0.137 to −0.029]; β_10–20mmHg_ = −0.103 [−0.156 to −0.05]; β_≥__20mmHg_ = −0.154 [−0.216 to −0.092], corresponding to 21.2% [7.4%–35%], 26.3% [13.1%–39.5%], and 39.4% [24.2%–54.5%] less progression relative to mean baseline SVD burden, respectively)—Fig. 3. The test for trend was also significant (p < 0.001), consistent with a dose–response relationship.

### BP mediation of the treatment assignment effect

The effect of treatment assignment on SVD burden change was mediated by SBP change (Supplemental Figure S3), as evidenced by the non-significant direct effect TreatmentGroup→SVDChange (β = −0.025 [−0.067 to 0.015]) and the significant indirect effect TreatmentGroup→ΔSBP→SVDChange (−0.04 [−0.06 to −0.023]).

## Discussion

In this post-hoc analysis of the SPRINT MRI substudy, intensive BP control to a systolic target of <120 mmHg was associated with less progression of SVD burden compared with a standard SBP target of <140 mmHg. Notably, we observed a graded, dose–response relationship between attained SBP reduction and longitudinal SVD burden change, supporting a biologically coherent link between hemodynamic exposure and microvascular brain injury and strengthening the plausibility of a causal effect of BP control on SVD progression.

While dose–response associations between SBP and cardiovascular morbidity and mortality are well established, such a relationship has not previously been demonstrated for SVD. A meta-analysis of large-scale BP-lowering randomized trials demonstrated relative risk reductions for major cardiovascular disease events, stroke, heart failure, and all-cause mortality that were proportional to the magnitude of the mean achieved BP reduction.^2^ Similarly, there is strong observational evidence of a continuous log–linear association between BP and vascular mortality extending down to at least 115/75 mmHg with no apparent threshold.^4^ The present results expand these prior findings by demonstrating, for the first time, a dose–response relationship between attained SBP reduction and SVD burden change, paralleling associations observed with other cardiovascular outcomes. Clinical trial results strongly support a SBP goal of <130 mmHg and, when feasible, <120 mmHg for cardiovascular disease risk reduction, and these goals have been incorporated in current guidelines for hypertension management.^36^ Our findings provide evidence that (i) the benefits of lower BP goals may extend to SVD burden, (ii) the magnitude of attained BP reduction may be important as incremental decreases in SBP were associated with gradually more favorable changes in SVD burden, and (iii) even modest SBP reductions of up to 10 mmHg could conceivably confer some benefit in slowing SVD progression for individuals unable to tolerate more aggressive BP drops.

Effective strategies for primary and secondary prevention of SVD are paramount for several reasons. First, most SVD cases are sporadic, making management of modifiable risk factors a logical and crucial target for prevention.^1^ Second, clinical manifestations of SVD are broad and include stroke (25% of ischemic and most hemorrhagic strokes), cognitive impairment (the predominant vascular contributor to cognitive decline and dementia), gait and balance problems, mood disorders, and extrapyramidal symptoms, substantially impacting quality of life and increasing disability burden and mortality.^1^^,^^37^ Third, many affected individuals are asymptomatic, creating a critical window of opportunity to implement preventive strategies before overt clinical manifestations develop.^1^ For these reasons, there is a pressing need for methods that can accurately capture the global SVD burden to identify individuals at risk and assess the efficacy of candidate preventive and therapeutic interventions (Supplemental Discussion 1).

The study of BP control in relation to SVD has almost exclusively focused on associations with individual MRI markers, such as WMH, PVS, and DTI indices.^5^, ^6^, ^7^, ^8^, ^9^^,^^38^ While prior investigations have provided valuable insights, relying on a single MRI measure to capture a complex and multifaceted biological process such as SVD poses considerable limitations. First, biological processes other than SVD—such as brain aging or neurodegeneration—can give rise to or influence the aforementioned MRI markers,^12^, ^13^, ^14^, ^15^, ^16^ making it difficult to distinguish the extent to which observed associations are attributable to SVD versus the effects of BP on other physiological or pathological processes. Second, individual MRI markers may only capture certain aspects of SVD rather than reflect its total burden.^1^^,^^17^^,^^18^ Finally, when relying on a single marker, measurement error is an important consideration that cannot be assessed or controlled for.

Considering these limitations, we previously introduced a measurement framework for SVD that, rather than relying on individual MRI measures, conceptualizes SVD as a latent (i.e., not directly observable) process measurable by a set of complementary imaging endophenotypes representing its sequelae. This latent variable framework offers advantages in both measurement properties and biological relevance; it explicitly accounts for measurement error, improves composite reliability by integrating multiple MRI markers, and better aligns with SVD neurobiology (Supplemental Discussion 2). Herein we applied this measurement framework to an independent cohort, where model fit indices supported its external validity. Furthermore, we evaluated the longitudinal measurement properties of its MRI indicators. These measurement properties were found to be conditional on age, further supporting the hypothesis that individual MRI markers are multidimensional—reflecting not only SVD but also aging-related processes.

Overall, this study represents a substantial advance over prior neuroimaging analyses by demonstrating a clear association between intensive BP control and total SVD burden using a novel, biology-informed biomarker that integrates three complementary imaging endophenotypes of vascular brain injury: periventricular WMH, basal ganglia PVS, and WM free water. In contrast to earlier SPRINT reports that examined surrogate markers of SVD, such as WMH volume, in isolation, our analysis captured a more pronounced reduction in SVD progression with intensive BP treatment (standardized mean difference of −0.4, compared with approximately −0.3 in prior WMH-based analyses).^5^ Perhaps even more importantly, we identified a clear dose–response pattern, whereby even modest SBP reductions of ≤10 mmHg conferred measurable neuroprotection, with progressively greater benefits for larger reductions.

This study also has limitations. First, this was a post-hoc analysis of a randomized clinical trial and change in overall SVD burden was not a prespecified endpoint. Second, follow-up MRI completion rate was lower than expected, in part due to the early termination of the intervention, which might have introduced attrition bias. However, baseline characteristics were overall similar between participants who completed only the baseline scan and those who underwent both baseline and follow-up scans (Table 1). Furthermore, sensitivity analyses including all individuals with available baseline MRI data yielded similar results, mitigating concerns about attrition bias. Third, as with other interventional studies, SPRINT is susceptible to cointervention bias. The fact that the association between treatment assignment and SVD progression was mediated by intervention-related changes in SBP decreases—but not entirely eliminates—the possibility that other, non-BP-related factors may have contributed to the observed treatment effect. Fourth, the dose–response analysis was based on post-randomization achieved SBP reduction and, because it does not preserve the randomization framework, it should be interpreted as observational rather than causal. Fifth, SPRINT did not include individuals with baseline diabetes, stroke, symptomatic heart failure, dementia, advanced kidney disease, or nursing home residents; therefore, generalizability of the present findings to these hypertensive populations remains uncertain and warrants further study. Sixth, other SVD endophenotypes such as lacunes and cerebral microbleeds were not considered in this analysis due to lack of required MRI sequences (cerebral microbleeds) or differences in ascertainment methods (lacunes). Finally, SPRINT was designed to test two different BP treatment goals in hypertensive individuals; therefore, this study cannot provide insights into the effects of hypertension treatment compared with uncontrolled hypertension or the relative effects of specific antihypertensive medications on the evolution of SVD burden.

To summarize, in this post-hoc analysis of a randomized clinical trial including hypertensive adults, targeting an SBP of <120 mmHg, compared with <140 mmHg, was associated with less progression of SVD burden. In addition, progressively greater reductions in achieved SBP were associated with incrementally more favorable changes in SVD burden, consistent with a clear dose–response relationship.

## Contributors

SC: conceptualization, formal analysis, investigation, methodology, software, visualisation, writing—original draft; NMP: conceptualization, data curation, investigation, methodology, writing—review & editing; LRP: investigation, methodology, writing—review & editing; NHH: investigation, methodology, writing—review & editing; TR: formal analysis, investigation, methodology, software, visualisation, writing—review & editing; DW: investigation, methodology, writing—review & editing; YZ: investigation, methodology, writing—review & editing; NSK: investigation, methodology, writing—review & editing; RNB: conceptualization, data curation, investigation, methodology, writing—review & editing; KCK: data curation, investigation, methodology, writing—review & editing; BCD: investigation, methodology, writing—review & editing; SS: investigation, methodology, writing—review & editing; IN: conceptualization, data curation, investigation, methodology, supervision, writing—review & editing; LL: conceptualization, data curation, investigation, methodology, supervision, writing—review & editing; CD: conceptualization, data curation, investigation, methodology, supervision, writing—review & editing; JDW: conceptualization, investigation, methodology, project administration, supervision, writing—review & editing; MH: conceptualization, investigation, methodology, project administration, supervision, writing—review & editing.

SC, NMP, TR, and MH directly accessed and verified the data reported in the manuscript.

## Data sharing statement

Deidentified participant data, along with relevant data dictionaries, are available through BioLINCC (https://biolincc.nhlbi.nih.gov/studies/sprint) to investigators who provide an Institutional Review Board/Ethics approval, or certification of exemption from Institutional Review Board/Ethics review, and who agree to the terms and conditions of a data use agreement. Data are available for any purpose, unless prohibited by the informed consent. The source code for the conducted analyses is available at https://github.com/UTHSCSA-NAL/SPRINT_SVD.

## Declaration of interests

NMP reports grants or contracts from the NIA, NHLBI, PCORI, and NCATS and participation on a Data Safety Monitoring Board or Advisory Board for the NIA and Astellas Pharmaceuticals. RNB reports grants or contracts from the NIH and participation on a Data Safety Monitoring Board or Advisory Board for ECOG-ACRIN. BCD reports royalties or licenses from Cambridge University Press, Elsevier, and Oxford University Press; consulting fees from Arkuda, Biogen, CervoMed, Eisai, Lantheus, Lilly, Merck, Novo Nordisk, and Quanterix; and participation on a Data Safety Monitoring Board or Advisory Board for Biogen and Lilly. SS reports other financial or non-financial interests with Novo Nordisk. IN reports grants or contracts from the NIH and consulting fees from Premier and Subtle Medical. MH reports grants or contracts from the NIH. All other authors report no disclosures relevant to the present manuscript.
