# Supplementary material for: Intensive versus standard blood pressure control and overall brain small vessel disease burden: a post-hoc analysis of the SPRINT randomized clinical trial
Source: eClinicalMedicine. 2026 Aug 11;99:104143. doi: 10.1016/j.eclinm.2026.104143 (PMC13482511; doi:10.1016/j.eclinm.2026.104143)
Supplement: Supplementary file 2 [file mmc2.docx]

**Supplementary Material. Table of Contents**

**Supplemental Table S1.** Scanner model by MRI site

**Supplemental Table S2.** MRI scanner acquisition parameters

**Supplemental Table S3.** Baseline characteristics of participants in the MRI substudy versus remaining SPRINT participants

**Supplemental Table S4.** Fit indices for SVD measurement models testing different levels of longitudinal measurement invariance

**Supplemental Table S5.** Parameter estimates for the covariance structure of the SVD measurement model (model 9 in Supplemental Table S4)

**Supplemental Table S6.** Parameter estimates for the mean structure of the SVD measurement model (model 9 in Supplemental Table S4)

**Supplemental Table S7.** Participant baseline characteristics across attained systolic blood pressure groups

**Supplemental Figure S1.** Multiple Indicators Multiple Causes (MIMIC) models examining age as a potential source of differential item functioning (DIF) across time

**Supplemental Figure S2.** Representative MRI examples of small vessel disease (SVD) indicator progression

**Supplemental Figure S3.** Multiple-indicator latent change score model examining mediation of the treatment assignment effect on small vessel disease (SVD) burden via systolic blood pressure (SBP) change

**Supplemental Methods 1.** Invariance testing strategy and differential item functioning investigation

**Supplemental Methods 2.** Multiple-indicator Latent Change Score (LCS) models

**Supplemental Methods 3.** Full information maximum likelihood with auxiliary variables

**Supplemental Results 1.** Measurement invariance testing and differential item functioning investigation

**Supplemental Discussion 1.** Prior efforts to capture global small vessel disease burden

**Supplemental Discussion 2.** Development of the small vessel disease measurement model

**Supplemental References**

| **Supplemental Table S1: Scanner model by MRI site.** | |
| --- | --- |
| **MRI Site** | **Scanner model** |
| University of Alabama at Birmingham | 3T Philips Achieva 3.2 |
| Boston University |  |
| Vanderbilt University |  |
| University of Miami | 3T Siemens TIM Trio VB17 |
| University of Pennsylvania |  |
| Wake Forest University | 3T Siemens Skyra VD11B |
| Case Western University | 3T Siemens Verio VB17 |

| **Supplemental Table S2: MRI scanner acquisition parameters.** | | | | | | | |
| --- | --- | --- | --- | --- | --- | --- | --- |
|  | **TR (ms)** | **TE (ms)** | **FOV (mm)** | **Slice thickness (mm)** | **Number of slices** | **Matrix** | **Scan duration (sec)** |
| T1-weighted | 1900 | 2.89 | 250 | 1 | 176 | 256 x 256 | 266 |
| T2-weighted | 3200 | 409 | 250 | 1 | 176 | 256 x 256 | 248 |
| FLAIR | 6000  (TI = 2200) | 160 | 250 | 1 | 160 | 258 x 221 | 500 |
| DTI | 7300 | 84 | 245 | 2.2 | 64 | 128 x 128 | 277 |
| Abbreviations: TR, repetition time; TE, echo time; TI, inversion time; FOV, field of view; FLAIR, fluid-attenuated inversion recovery; DTI, diffusion tensor imaging. | | | | | | | |

| **Supplemental Table S3: Baseline characteristics of participants in the MRI substudy versus remaining SPRINT participants.** | | | | |
| --- | --- | --- | --- | --- |
|  | | In MRI substudy  (n = 670) | Not in MRI substudy  (n = 8,688 ^a^) | P value |
| Randomized to intensive treatment, n (%) | | 355 (53.0) | 4322 (49.7) | 0.12 |
| Age, mean (SD), years | | 67.3 (8.2) | 68.0 (9.5) | 0.08 |
| Sex, female, n (%) | | 271 (40.4) | 3061 (35.2) | 0.007 |
| Race/ethnicity, n (%) | Black | 218 (32.5) | 2584 (29.7) | <0.001 |
|  | Hispanic ^b^ | 36 (5.4) | 948 (10.9) |  |
|  | White | 406 (60.6) | 4990 (57.4) |  |
|  | Other ^c^ | 10 (1.5) | 166 (1.9) |  |
| Education, n (%) | Less than high school | 53 (7.9) | 823 (9.5) | 0.39 |
|  | High school graduate | 103 (15.4) | 1433 (16.5) |  |
|  | Post high school training | 239 (35.7) | 3072 (35.4) |  |
|  | College graduate or greater | 275 (41.0) | 3360 (38.7) |  |
| Smoking status, n (%) | Never smoker | 301 (44.9) | 3846 (44.3) | 0.94 |
|  | Former smoker | 282 (42.1) | 3690 (42.5) |  |
|  | Current smoker | 87 (13.0) | 1152 (13.3) |  |
| Polypharmacy | <5 medications | 289 (43.1) | 3783 (43.5) | 0.32 |
|  | 5-9 medications | 274 (40.9) | 3696 (42.5) |  |
|  | ≥10 medications | 107 (16.0) | 1209 (13.9) |  |
| Body mass index, mean (SD), kg/m^2^ | | 29.8 (5.4) | 29.9 (5.8) | 0.70 |
| Fasting HDL cholesterol, mean (SD), mg/dL | | 53.5 (14.5) | 52.8 (14.5) | 0.22 |
| History of CVD, n (%) | | 93 (13.9) | 1784 (20.5) | <0.001 |
| Systolic blood pressure, mean (SD), mmHg | | 138.0 (16.6) | 139.8 (15.5) | 0.004 |
| Diastolic blood pressure, mean (SD), mmHg | | 77.9 (11.4) | 78.1 (12.0) | 0.54 |
| Orthostatic hypotension ^d^, n (%) | | 43 (6.4) | 642 (7.4) | 0.39 |
| eGFR ^e^, mean (SD), mL/min/1.73m^2^ | | 72.3 (20.6) | 71.7 (20.6) | 0.50 |
| Log urine albumin-to-creatinine ratio, mean (SD),  log(mg/g) | | 2.6 (1.2) | 2.6 (1.2) | 0.90 |
| Serum bicarbonate, mean (SD), mmol/L | | 26.5 (2.5) | 26.3 (2.6) | 0.13 |
| Abbreviations: HDL, high-density lipoprotein; CVD, cardiovascular disease; eGFR, estimated glomerular filtration rate.  ^a^ Excludes 3 participants without a measured white matter lesion volume at baseline, but who completed baseline MRI scan with a measurement of total brain volume that passed quality control.  ^b^ Hispanic race/ethnicity encompasses a self-report of being of Spanish, Hispanic, or Latino origin, independent of any other race/ethnicity designation.  ^c^ Other race/ethnicity includes categories of Asian, American Indian/Alaska Native, Native Hawaiian/Pacific Islander, or other.  ^d^ Defined as a standing systolic blood pressure minus seated systolic blood pressure ≤-20 mmHg, or a standing diastolic blood pressure minus seated diastolic blood pressure ≤-10 mmHg.  ^e^ Based on the 4-variable Modification of Diet in Renal Disease equation. | | | | |

| **Supplemental Table S4: Fit indices for SVD measurement models testing different levels of longitudinal measurement invariance.** | | | | | |  |
| --- | --- | --- | --- | --- | --- | --- |
| **Model** | **Chi-square scaled ^a^** | **P value scaled ^a^** | **Robust**  **CFI ^a^** | **Robust**  **RMSEA ^a^** | **SRMR** |  |
|  | *LCFA models not accounting for DIF* | | | | |  |
| 1. Configural invariance | 7.935 | 0.160 | 0.999 | 0.035 | 0.012 | Raw  SVD indicators |
| 2. Metric invariance | 34.370 | <0.001 | 0.988 | 0.093 | 0.039 |  |
| 3. Scalar invariance | 114.733 | <0.001 | 0.953 | 0.165 | 0.114 |  |
| 4. Configural invariance | 7.941 | 0.159 | 0.999 | 0.035 | 0.012 | ROI volume-normalized SVD  indicators |
| 5. Metric invariance | 24.470 | 0.001 | 0.992 | 0.074 | 0.032 |  |
| 6. Scalar invariance | 108.52 | <0.001 | 0.958 | 0.153 | 0.097 |  |
|  | *LCFA models accounting for DIF due to age* | | | | |  |
| 7. Configural invariance | 8.876 | 0.353 | 1.000 | 0.016 | 0.013 | Raw  SVD  indicators |
| 8. Metric invariance | 23.269 | 0.010 | 0.995 | 0.055 | 0.023 |  |
| 9. Scalar invariance | 26.974 | 0.008 | 0.994 | 0.053 | 0.024 |  |
| 10. Configural invariance | 9.576 | 0.296 | 0.999 | 0.021 | 0.011 | ROI volume-normalized  SVD indicators |
| 11. Metric invariance | 17.846 | 0.058 | 0.997 | 0.042 | 0.019 |  |
| 12. Scalar invariance | 22.042 | 0.037 | 0.996 | 0.043 | 0.019 |  |
| Abbreviations: SVD, small vessel disease; LCFA, longitudinal confirmatory factor analysis; DIF, differential item functioning; CFI, comparative fit index; RMSEA, root mean square error of approximation; SRMR, standardized root mean square residual.  ^a^ Test statistics and fit indices that are robust to non-normality have been calculated based on a scaled test statistic that is asymptotically equivalent to the Yuan-Bentler T2* test statistic.^1^ | | | | | | |

| **Supplemental Table S5: Parameter estimates for the covariance structure of the SVD measurement model (model 9 in Supplemental Table S4).** | | | | | | |
| --- | --- | --- | --- | --- | --- | --- |
| **Parameter** | **Baseline (t0)** | | | **Follow-up (t1)** | | |
|  | **Unstandardized**  **Estimate** | **Standard Error** | **Standardized**  **Estimate** | **Unstandardized**  **Estimate** | **Standard Error** | **Standardized**  **Estimate** |
| *Equality-constrained estimates* | | | | | | |
| **Loadings**  Periventricular WMH  WM free water  Basal ganglia PVS | 1  0.562  0.356 | 0.074  0.064 | 0.814  0.545  0.373 | 1  0.562  0.356 | 0.074  0.064 | 0.756  0.586  0.449 |
| *Unconstrained estimates* | | | | | | |
| ***Baseline age coefficients***  Periventricular WMH  WM free water  Basal ganglia PVS | 0.018  0.025  0.006 | 0.003  0.002  0.002 | 0.323  0.550  0.137 | 0.024  0.027  0.004 | 0.004  0.002  0.002 | 0.332  0.509  0.094 |
| **Follow-up time coefficients**  Periventricular WMH  WM free water  Basal ganglia PVS |  |  |  | 0.039  0.043  -0.009 | 0.032  0.018  0.012 | 0.022  0.033  -0.008 |
| **Residual variances**  Periventricular WMH  WM free water  Basal ganglia PVS | 0.049  0.060  0.107 | 0.019  0.007  0.009 | 0.233  0.401  0.842 | 0.117  0.077  0.103 | 0.031  0.010  0.009 | 0.322  0.405  0.789 |
| **Factor variances**  SVD | 0.139 | 0.027 | 1 | 0.207 | 0.039 | 1 |
| **Covariance**  SVD_t0 ↔ SVD_t1 | 0.159 | 0.031 | 0.937 | 0.159 | 0.031 | 0.937 |
| Abbreviations: SVD, small vessel disease; WMH, white matter hyperintensities; WM, white matter; PVS, perivascular spaces. | | | | | | |

| **Supplemental Table S6: Parameter estimates for the mean structure of the SVD measurement model (model 9 in Supplemental Table S4).** | | | | | | |
| --- | --- | --- | --- | --- | --- | --- |
| **Parameter** | **Baseline (t0)** | | | **Follow-up (t1)** | | |
|  | **Unstandardized**  **Estimate** | **Standard Error** | **Standardized**  **Estimate** | **Unstandardized**  **Estimate** | **Standard Error** | **Standardized**  **Estimate** |
| *Equality-constrained estimates* | | | | | | |
| **Intercepts**  Periventricular WMH  WM free water  Basal ganglia PVS | 0.391  2.325  0.968 | 0.021  0.015  0.017 | 0.852  6.036  2.714 | 0.391  2.325  0.968 | 0.021  0.015  0.017 | 0.649  5.324  2.680 |
| **Factor means**  SVD_t0 | 0 |  |  |  |  |  |
| *Unconstrained estimates* | | | | | | |
| **Factor means**  SVD_t1 |  |  |  | -0.042 | 0.122 | -0.092 |
| Abbreviations: SVD, small vessel disease; WMH, white matter hyperintensities; WM, white matter; PVS, perivascular spaces. | | | | | | |

| **Supplemental Table S7: Participant baseline characteristics across attained systolic blood pressure groups.** | | | | | | |
| --- | --- | --- | --- | --- | --- | --- |
|  | | SBP increase  (n = 118) | SBP drop 0-10 mmHg  (n = 106) | SBP drop 10-20 mmHg  (n = 103) | SBP drop ≥ 20 mmHg  (n = 114) | P value |
| *Demographic and vascular characteristics* | | | | | |  |
| Age, mean (SD), years | | 67.2 (7.3) | 68.8 (9.5) | 66.9 (7.2) | 69.9 (9.1) | 0.03 |
| Sex, n (%) | Men | 70 (59%) | 72 (68%) | 66 (64%) | 71 (62%) | 0.60 |
|  | Women | 48 (41%) | 34 (32%) | 37 (36%) | 43 (38%) |  |
| Race/ethnicity, n (%) | Black | 37 (31%) | 30 (28%) | 31 (30%) | 24 (21%) | 0.27 |
|  | Hispanic | 8 (6.8%) | 2 (1.9%) | 6 (5.8%) | 5 (4.4%) |  |
|  | White | 71 (60%) | 72 (68%) | 63 (61%) | 85 (75%) |  |
|  | Other | 2 (1.7%) | 2 (1.9%) | 3 (2.9%) | 0 (0%) |  |
| Fasting HDL cholesterol, mean (SD), mg/dL | | 53.3 (15.0) | 54.1 (17.4) | 51.6 (13.2) | 54.3 (16.1) | 0.57 |
| Body mass index, mean (SD), kg/m^2^ | | 29.7 (4.7) | 29.1 (5.3) | 29.6 (5.3) | 29.0 (4.7) | 0.68 |
| Systolic blood pressure, mean (SD), mmHg | | 123.9 (10.6) | 133.6 (10.4) ^a^ | 137.7 (10.8) ^a^ | 154.7 (14.3) ^a,b,c^ | <0.001 |
| Diastolic blood pressure, mean (SD), mmHg | | 72.0 (9.8) | 75.4 (11.0) | 78.2 (9.9) ^a^ | 83.9 (11.9) ^a,b,c^ | <0.001 |
| *MRI-derived volumes* | | | | | |  |
| Intracranial volume, mean (SD), mm^3^ | | 1,404,027 (139,982) | 1,413,366 (134,251) | 1,405,982 (135,460) | 1,411,349 (141,693) | 0.95 |
| Periventricular WM ROI, mean (SD), mm^3^ | | 118,632.2 (20,247.2) | 120,820.4 (20,982.7) | 117,717.0 (23,148.7) | 124,350.0 (21,712.0) | 0.10 |
| Basal ganglia ROI, mean (SD), mm^3^ | | 27,277.2 (3,010.4) | 26,955.7 (2,717.8) | 26,996.7 (2,905.6) | 26,854.2 (3,185.5) | 0.73 |
| *SVD indicators* | | | | | |  |
| Periventricular WMH, median (IQR), mm^3^ | | 2,303.6 (1,104.3, 4,349.0) | 2,267.3 (997.6, 4,456.3) | 1,768.0 (920.0, 4,262.8) | 2,731.7 (1,277.5, 6,852.4) ^c^ | 0.02 |
| Mean WM free water, mean (SD) | | 0.229 (0.037) | 0.235 (0.038) | 0.223 (0.038) | 0.242 (0.039) ^c^ | 0.002 |
| Basal ganglia PVS, mean (SD), count | | 98.3 (36.3) | 95.1 (34.0) | 90.9 (33.9) | 102.6 (40.2) | 0.11 |
| *ROI volume-normalized SVD indicators* | | | | | |  |
| Periventricular WMH, median (IQR), mm^3^/mm^3^ | | 0.020 (0.009, 0.038) | 0.019 (0.009, 0.034) | 0.014 (0.009, 0.033) | 0.024 (0.012, 0.052) ^c^ | 0.02 |
| Basal ganglia PVS, mean (SD), count/mm^3^ | | 0.004 (0.001) | 0.004 (0.001) | 0.003 (0.001) | 0.004 (0.001) ^c^ | 0.05 |
| *Age-adjusted SVD indicators* | | | | | | |
| Periventricular WMH, median (IQR) | | -1,225.0 (-2,605.9, 509.1) | -1,226.3 (-2,550.5, 522.6) | -1,467.7 (-2,477.4, 217.1) | -893.5 (-2,680.8, 2,437.3) | 0.37 |
| Mean WM free water, mean (SD) | | -0.001 (0.034) | 0.001 (0.032) | -0.006 (0.029) | 0.006 (0.034) | 0.06 |
| Basal ganglia PVS, mean (SD) | | 2.0 (36.7) | -2.2 (33.8) | -5.2 (33.6) | 4.7 (39.2) | 0.18 |
| ROI volume-normalized periventricular WMH, median (IQR) | | -0.009 (-0.019, 0.004) | -0.011 (-0.019, 0.006) | -0.012 (-0.019, 0.003) | -0.007 (-0.020, 0.022) | 0.27 |
| ROI volume-normalized basal ganglia PVS, mean (SD) | | 0.0000 (0.0012) | -0.0001 (0.0011) | -0.0002 (0.0011) | 0.0002 (0.0012) | 0.12 |
| Abbreviations: SBP, systolic blood pressure; HDL, high-density lipoprotein; WM, white matter; ROI, region of interest; SVD, small vessel disease; WMH, white matter hyperintensities; PVS, perivascular spaces.  ^a, b, c^ significantly different compared to the value in the first (SBP increase), second (SBP drop 0-10 mmHg), and third (SBP drop 10-20 mmHg) attained systolic blood pressure group after Bonferroni correction for multiple comparisons. | | | | | | |

**Supplemental Figure S1: Multiple Indicators Multiple Causes (MIMIC) models examining age as a potential source of differential item functioning (DIF) across time.**

Small vessel disease (SVD) is modeled as a latent construct, measured by periventricular white matter hyperintensities (WMH), white matter (WM) free water, and basal ganglia perivascular spaces (PVS) at baseline (t0) and follow-up (t1). Direct effects from follow-up time (to follow-up MRI indicator measurements – t1) and from baseline age (to both baseline and follow-up MRI indicator measurements – t0 and t1) are tested for each MRI indicator while accounting for the SVD-mediated indirect age effects. A statistically significant direct effect would suggest age as a source of DIF for the respective MRI indicator. Asterisks indicate statistically significant parameter estimates at p <.05 (*), p <.01 (**), and p <.001 (***).

**A. MIMIC model for periventricular white matter hyperintensities (WMH)**

**B. MIMIC model for mean white matter (WM) free water**

**C. MIMIC model for basal ganglia perivascular spaces (PVS)**

**Supplemental Figure S2: Representative MRI examples of small vessel disease (SVD) indicator progression.**

Representative MRI examples of periventricular white matter hyperintensity (PWMH) and basal ganglia perivascular space (BGPVS) progression are shown for two participants: one from the standard blood pressure (BP) treatment group with a high latent SVD change score, reflecting greater overall SVD burden progression, and one from the intensive BP treatment group with a low latent SVD change score, reflecting smaller overall SVD burden progression.

A. **PWMH progression**

B. **BGPVS progression**

**Supplemental Figure S3:** **Multiple-indicator latent change score model examining mediation of the treatment assignment effect on small vessel disease (SVD) burden via systolic blood pressure (SBP) change.**

SVD is modeled as a latent construct, measured by periventricular white matter hyperintensities (WMH), white matter (WM) free water, and basal ganglia perivascular spaces (PVS) at baseline (t0) and follow-up (t1). The model includes direct effects of baseline age and follow-up time on each MRI indicator to account for age-related differential item functioning. Latent SVD change is defined as the difference in SVD between t1 and t0. Intensive BP treatment group membership is modeled as a predictor of latent SVD change, with change in SBP from baseline (ΔSBP) specified as a potential mediator. Arrow values represent regression coefficients (or covariances for double-headed arrows). Asterisks indicate statistically significant parameter estimates at p <.05 (*), p <.01 (**), and p <.001 (***).

# **Supplemental Methods 1: Invariance testing strategy and differential item functioning investigation**

Measurement invariance was assessed in three hierarchically nested steps. First, configural invariance was tested, assuming only same factor structure across time. Second, metric invariance was tested by imposing equality constraints on the MRI indicator loadings across time. Lastly, scalar invariance was tested by additionally constraining the MRI indicator intercepts to be equal across time. We used the commonly accepted criterion of decreases in comparative fit index (CFI) of >0.01 (ΔCFI <-0.01) to determine whether the null hypothesis of invariance should be rejected.^2^

In cases where invariance was not supported, we used Multiple Indicators Multiple Causes (MIMIC) models to examine conceptually plausible sources of differential item functioning (DIF) over time. In these models, a direct effect from the covariate of interest to both the small vessel disease (SVD) factor and the indicator under evaluation was estimated, and DIF was determined to be present when the direct relationship between the covariate and the indicator in question was statistically significant.^3,4^ Once potential sources of DIF were identified, we conditioned the longitudinal confirmatory factor analysis (LCFA) models on these covariates and repeated the measurement invariance testing sequence.

# **Supplemental Methods 2: Multiple-indicator Latent Change Score (LCS) models**

We used multiple-indicator Latent Change Score (LCS) models to examine the effect of treatment assignment on small vessel disease (SVD) burden. In this model, SVD burden for individual $i$ at time $t$ is a deterministic function of SVD burden at a previous timepoint $(t-1)$ and a latent change score (ΔSVD – the difference between SVD burden measured at follow-up and baseline).

Mathematically,

${SVD}_{[t]i}={SVD}_{[t-1]i}+\Delta{SVD}_{i}$ *(1)*

Where ${SVD}_{[t]i}$ is measured by the following measurement model,

$X_{[t]ij}={a_{j}+\lambda}_{j}{SVD}_{[t]i}+\varepsilon_{[t]ij}$ *(2)*

Where $X_{[t]ij}$ is the observed value of MRI indicator $j$ for individual $i$ at time $t$, $a_{j}$ is the indicator intercept, $\lambda_{j}$ is the indicator loading on the latent SVD factor, and $\varepsilon_{[t]ij}$ is the measurement error.

In equation (1), the following constraints were imposed: (i) the residual variance and intercept of ${SVD}_{[t]i}$were fixed to zero as ${SVD}_{[t]i}$is fully determined by ${SVD}_{[t-1]i}$ and $\Delta{SVD}_{i}$, and (ii) the autoregressive coefficient (path from ${SVD}_{[t-1]i}$ to ${SVD}_{[t]i}$) and the latent change factor loading (path from $\Delta{SVD}_{i}$ to ${SVD}_{[t]i}$) were fixed to one.

In equation (2), the following constraints were imposed: (i) MRI indicator loadings $\lambda_{j}$ were constrained to be equal across time (i.e., metric invariance), (ii) MRI indicator intercepts $a_{j}$ were constrained to be equal across time (i.e., scalar invariance), and (iii) identifiability constraints ($\lambda_{1}$ [corresponding to periventricular white matter hyperintensity indicator loading] was fixed to 1, and $a_{1}$ [corresponding to periventricular white matter hyperintensity indicator intercept] was fixed to 0).

$\Delta{SVD}_{i}$ and ${SVD}_{[t-1]i}$ were allowed to covary considering that baseline SVD burden and SVD burden change might be correlated.

In cases of DIF, we modified the LCS model by including regressions of the MRI indicators on covariates accounting for DIF, conditioning the measurement model on plausible values of these covariates.

For the conditional LCS model, measurement equation (2) is modified as follows:

$X_{[t]ij}={a_{j}+\lambda}_{j}{SVD}_{[t]i}+\beta_{\left[ t \right]j}C_{\left[ t \right]i}+ \varepsilon_{[t]ij}$ (3)

Where C is the covariate accounting for DIF for individual $i$ at time $t$, and $\beta_{\left[ t \right]j}$ denotes its direct effect on MRI indicator $j$.

# **Supplemental Methods 3: Full information maximum likelihood with auxiliary variables**

In the context of full information maximum likelihood (FIML) estimation, auxiliary variables are variables that correlate either with the pattern of missingness or with variables that have missing values. Auxiliary variables can be incorporated into an FIML-based analysis to improve estimation of parameters and standard errors in the presence of missing data, without altering the model of primary interest.^5^

Consistent with prior analyses of SPRINT brain MRI data,^6-8^ we included the following set of auxiliary variables: sex, race/ethnicity (White, Black, Hispanic, or Other), education (less than high school education, high school graduate, additional training beyond high school but no college degree, college graduate or higher), polypharmacy (<5 medications, 5 to 9 medications, or ≥10 medications), smoking status (never, former, or current smoker), history of cardiovascular disease (present or absent), systolic blood pressure, diastolic blood pressure, body mass index, fasting high-density lipoprotein cholesterol, serum bicarbonate, estimated glomerular filtration rate, log urine albumin-to-creatinine ratio, Logical Memory delayed recall score, and baseline intracranial volume.

# **Supplemental Results 1: Measurement invariance testing and differential item functioning investigation**

The longitudinal confirmatory factor analysis (LCFA) model assuming configural invariance demonstrated excellent fit with either raw or ROI volume-normalized MRI indicators (Supplemental Table S4). However, when higher levels of measurement invariance were imposed, the model fit progressively deteriorated, particularly under scalar invariance constraints. We therefore explored age as a potential source of differential item functioning (DIF), considering it could influence the MRI indicators independently of small vessel disease – SVD (i.e., through neurodegenerative or other age-related pathways^9-14^). Indeed, Multiple Indicators Multiple Causes (MIMIC) models revealed significant direct effects of age on each SVD indicator at baseline and/or follow-up (Supplemental Figure S1), indicating that age contributed to measurement non-invariance.

# **Supplemental Discussion 1: Prior efforts to capture global small vessel disease burden**

In cerebral small vessel disease (SVD) research, the challenge of developing an imaging scale summarizing SVD burden is not a new one. It was acknowledged in the Standards for Reporting Vascular Changes on Neuroimaging 1 (STRIVE-1)^15^ and reiterated in STRIVE-2.^16^ Motivations behind this challenge include the need to improve prediction of clinical outcomes as well as participant selection and stratification for intervention trials. Although some SVD scores have been proposed since this challenge was first described, these are based on visual rating scales.^17,18^ Among other groups, we have previously described the potential limitations of visual rating methods for SVD marker detection, including labor-intensity and impracticality of application to large-scale epidemiological datasets, reliance on semi-quantitative scales and ceiling effects, lack of regional information, and inter-/intra-rater variability concerns.^17,19-21^ Our approach was based on previously developed and validated state-of-the-art deep learning models that offer timely whole-brain and region-specific quantifications for various SVD endophenotypes.^22-24^ Additionally, in contrast to previous approaches, our analytical framework explicitly accounts and corrects for measurement error.

# **Supplemental Discussion 2: Development of the small vessel disease measurement model**

Leveraging prior evidence that links the spatial distribution of MRI markers to their underlying pathogenetic substrates, we identified a set of MRI measures in strategic brain regions that are more likely to reflect small vessel disease (SVD)-specific pathology as opposed to other biological processes.^25^ We then modeled SVD through a structural equation modeling/classical test theory framework, which allows the integration of multiple relevant SVD endophenotypes – each potentially capturing distinct aspects of the disease – into a single dimension, while also accounting for measurement error. This SVD latent factor model was developed and tested in the Multi-ethnic Study of Atherosclerosis (MESA), where we showcased its effectiveness in identifying mediating effects in the relationships of aging and cardiovascular disease risk with cognitive function – through pathways that both involve and are independent of brain atrophy. We further demonstrated that these mediating effects were not fully captured when considering individual MRI markers in isolation.

# **Supplemental References**

1.	Yuan K-H, Bentler PM. Three Likelihood-Based Methods For Mean and Covariance Structure Analysis With Nonnormal Missing Data. *Sociological Methodology*. 2000/01/01 2000;30(1):165-200. doi:<https://doi.org/10.1111/0081-1750.00078>

2.	Cheung GW, Rensvold RB. Evaluating Goodness-of-Fit Indexes for Testing Measurement Invariance. *Structural equation modeling*. 2002;9(2):233-255. doi:10.1207/S15328007SEM0902_5

3.	Finch H. The MIMIC Model as a Method for Detecting DIF: Comparison With Mantel-Haenszel, SIBTEST, and the IRT Likelihood Ratio. *Applied Psychological Measurement*. 2005/07/01 2005;29(4):278-295. doi:10.1177/0146621605275728

4.	Muthén BO. Latent variable modeling in heterogeneous populations. *Psychometrika*. 1989/09/01 1989;54(4):557-585. doi:10.1007/BF02296397

5.	Enders CK. A Note on the Use of Missing Auxiliary Variables in Full Information Maximum Likelihood-Based Structural Equation Models. *Structural Equation Modeling: A Multidisciplinary Journal*. 2008/07/29 2008;15(3):434-448. doi:10.1080/10705510802154307

6.	Nasrallah IM, Pajewski NM, Auchus AP, et al. Association of Intensive vs Standard Blood Pressure Control With Cerebral White Matter Lesions. *Jama*. Aug 13 2019;322(6):524-534. doi:10.1001/jama.2019.10551

7.	Dolui S, Detre JA, Gaussoin SA, et al. Association of Intensive vs Standard Blood Pressure Control With Cerebral Blood Flow: Secondary Analysis of the SPRINT MIND Randomized Clinical Trial. *JAMA Neurol*. Apr 1 2022;79(4):380-389. doi:10.1001/jamaneurol.2022.0074

8.	Rashid T, Li K, Toledo JB, et al. Association of Intensive vs Standard Blood Pressure Control With Regional Changes in Cerebral Small Vessel Disease Biomarkers: Post Hoc Secondary Analysis of the SPRINT MIND Randomized Clinical Trial. *JAMA Network Open*. 2023;6(3):e231055-e231055. doi:10.1001/jamanetworkopen.2023.1055

9.	Garnier-Crussard A, Bougacha S, Wirth M, et al. White matter hyperintensity topography in Alzheimer's disease and links to cognition. *Alzheimer's & Dementia*. 2022/03/01 2022;18(3):422-433. doi:<https://doi.org/10.1002/alz.12410>

10.	Tsai H-H, Pasi M, Tsai L-K, et al. Superficial Cerebellar Microbleeds and Cerebral Amyloid Angiopathy. 2020;51(1):202-208. doi:doi:10.1161/STROKEAHA.119.026235

11.	Alida AG, Alexandra S, Wiesje MvdF, et al. Heterogeneity of small vessel disease: a systematic review of MRI and histopathology correlations. *Journal of Neurology, Neurosurgery &amp; Psychiatry*. 2011;82(2):126. doi:10.1136/jnnp.2009.204685

12.	Habes M, Sotiras A, Erus G, et al. White matter lesions: Spatial heterogeneity, links to risk factors, cognition, genetics, and atrophy. *Neurology*. Sep 4 2018;91(10):e964-e975. doi:10.1212/wnl.0000000000006116

13.	Garnier-Crussard A, Cotton F, Krolak-Salmon P, Chételat G. White matter hyperintensities in Alzheimer's disease: Beyond vascular contribution. *Alzheimer's & Dementia*. 2023/08/01 2023;19(8):3738-3748. doi:<https://doi.org/10.1002/alz.13057>

14.	Bergamino M, Walsh RR, Stokes AM. Free-water diffusion tensor imaging improves the accuracy and sensitivity of white matter analysis in Alzheimer’s disease. *Scientific Reports*. 2021/03/26 2021;11(1):6990. doi:10.1038/s41598-021-86505-7

15.	Wardlaw JM, Smith EE, Biessels GJ, et al. Neuroimaging standards for research into small vessel disease and its contribution to ageing and neurodegeneration. *Lancet Neurol*. Aug 2013;12(8):822-38. doi:10.1016/S1474-4422(13)70124-8

16.	Duering M, Biessels GJ, Brodtmann A, et al. Neuroimaging standards for research into small vessel disease-advances since 2013. *Lancet Neurol*. Jul 2023;22(7):602-618. doi:10.1016/s1474-4422(23)00131-x

17.	Paradise MB, Shepherd CE, Wen W, Sachdev PS. Neuroimaging and neuropathology indices of cerebrovascular disease burden. *Neurology*. 2018/08/14 2018;91(7):310-320. doi:10.1212/WNL.0000000000005997

18.	Staals J, Makin SD, Doubal FN, Dennis MS, Wardlaw JM. Stroke subtype, vascular risk factors, and total MRI brain small-vessel disease burden. *Neurology*. Sep 30 2014;83(14):1228-34. doi:10.1212/wnl.0000000000000837

19.	Charisis S, Rashid T, Liu H, et al. Assessment of Risk Factors and Clinical Importance of Enlarged Perivascular Spaces by Whole-Brain Investigation in the Multi-Ethnic Study of Atherosclerosis. *JAMA Netw Open*. Apr 3 2023;6(4):e239196. doi:10.1001/jamanetworkopen.2023.9196

20.	Silva GD, Telles JPM, Rimkus CM, et al. Reliability and validity of the total cerebral small vessel disease score: a systematic review and meta-analysis. *Front Neurol*. 2025;16:1593402. doi:10.3389/fneur.2025.1593402

21.	Torres MF, Charisis S, Rashid T, et al. Comparative Analysis of AI-based Quantification vs. Visual Rating of Enlarged Perivascular Spaces in the MESA Cohort. *AJNR Am J Neuroradiol*. Dec 15 2025;doi:10.3174/ajnr.A9129

22.	Doshi J, Erus G, Habes M, Davatzikos C. DeepMRSeg: A convolutional deep neural network for anatomy and abnormality segmentation on MR images. *arXiv preprint arXiv:190702110*. 2019;

23.	Rashid T, Abdulkadir A, Nasrallah I, et al. DEEPMIR: a deep neural network for differential detection of cerebral microbleeds and iron deposits in MRI. *Scientific Reports*. 07/08 2021;11:14124. doi:10.1038/s41598-021-93427-x

24.	Rashid T, Liu H, Ware JB, et al. Deep learning based detection of enlarged perivascular spaces on brain MRI. *Neuroimage: Reports*. 2023/03/01/ 2023;3(1):100162. doi:<https://doi.org/10.1016/j.ynirp.2023.100162>

25.	Charisis S, Rashid T, Dintica C, et al. Assessing the Global Impact of Brain Small Vessel Disease on Cognition: The Multi-Ethnic Study of Atherosclerosis. *Alzheimer's & Dementia*. 2025/06/01 2025;21(6):e70326. doi:<https://doi.org/10.1002/alz.70326>
